# Selenocysteine induces apoptosis in human glioma cells: evidence for TrxR1-targeted inhibition and signaling crosstalk

**DOI:** 10.1038/s41598-017-06979-2

**Published:** 2017-07-25

**Authors:** Cun-dong Fan, Xiao-yan Fu, Zong-yong Zhang, Ming-zhi Cao, Jing-yi Sun, Ming-feng Yang, Xiao-ting Fu, Shi-jun Zhao, Lu-rong Shao, Hui-fang Zhang, Xiao-yi Yang, Bao-liang Sun

**Affiliations:** 1 Key Lab of Cerebral Microcirculation in Universities of Shandong, Taishan Medical University, Taian, Shandong 271000 China; 2 Department of Neurosurgery, Huxi Hospital, Jining Medical University, Shanxian, 274300 Shandong, China; 3 Wonju Severance Christian Hospital, Yonsei University Wonju College of Medicine, Wonju, Gangwon 220-701 Korea; 4 Department of Neurology, Affiliated Hospital of Taishan Medical University, Taian, 271000 Shandong China

## Abstract

Thioredoxin reductase (TrxR) as a selenium (Se)-containing antioxidase plays key role in regulating intracellular redox status. Selenocystine (SeC) a natural available Se-containing amino acid showed novel anticancer potential through triggering oxidative damage-mediated apoptosis. However, whether TrxR-mediated oxidative damage was involved in SeC-induced apoptosis in human glioma cells has not been elucidated yet. Herein, SeC-induced human glioma cell apoptosis was detected *in vitro*, accompanied by PARP cleavage, caspases activation and DNA fragmentation. Mechanically, SeC caused mitochondrial dysfunction and imbalance of Bcl-2 family expression. SeC treatment also triggered ROS-mediated DNA damage and disturbed the MAPKs and AKT pathways. However, inhibition of ROS overproduction effectively attenuated SeC-induced oxidative damage and apoptosis, and normalized the expression of MAPKs and AKT pathways, indicating the significance of ROS in SeC-induced apoptosis. Importantly, U251 human glioma xenograft growth in nude mice was significantly inhibited *in vivo*. Further investigation revealed that SeC-induced oxidative damage was achieved by TrxR1-targeted inhibition *in vitro* and *in vivo*. Our findings validated the potential of SeC to inhibit human glioma growth by oxidative damage-mediated apoptosis through triggering TrxR1-targeted inhibition.

## Introduction

Human glioma represents the most common type of human brain tumor, which the average survival period is not beyond 16 months^1–3^. Chemotherapy as one of the most effective ways for treatment of human glioma remains disappointed because of its high invasiveness, malignance and resistance^2–5^. Hence, searching novel agents with high efficiency and low side effects for combating human glioma is an urgent priority in drug development.

Selenium (Se) as a mineral microelement exhibits fundamental importance to human^6^. The chemotherapy and chemoprevention of Se-containing compounds have already been confirmed by the epidemiological studies, preclinical investigations and clinical trials^7–9^. Accumulated evidences have revealed that apoptosis is the major mechanism for anticancer action of Se-containing compounds^7^. Studies reported that Se-containing compounds could inhibit human cancer cells growth *in vitro* and *in vivo* by induction of cell apoptosis^5, 10–15^. Importantly, selenium compounds display less cytotoxicity towards human normal cells, indicating the novel selectivity between cancer cells and human normal cells^12^. Based on our pervious studies, generation of reactive oxide species (ROS), oxidative stress-mediated DNA damage, mitochondrial dysfunction, imbalance of Bcl-2 family expression, and the dysregulation of MAPKs and AKT pathways all contributed to Se-containing compounds-induced apoptosis in several human cancer cells^11–15^. We have emphasized the significance of ROS in Se-containing compounds-induced apoptosis in our previous reports^11–15^. However, the mechanism remains elusive, especially the source of ROS has not well investigated yet.

Se, including organic-Se, inorganic Se and Se-containing proteins, are all enzymatically or non-enzymatically metabolized in the biological environment, and finally incorporated into Se-containing proteins^16^. Se can function in the active sites of a large number of Se-containing enzymes, such as glutathione peroxidase (GSH-Px) and thioredoxin reductase (TrxR) ^17–19^. Selenocysteine as the major form found in Se-containing proteins plays important role in regulating the intracellular redox balance^16^. Se supplement either enhance the intracellular antioxidant ability by replenishing the Se-containing enzymes, or induce ROS-mediated cancer cell apoptosis through disturbing the antioxidase system, which depends on the form and dose of Se-containing compounds.

TrxR as a selenium-containing oxidoreductases is overpressed in many human tumors and is of significance in maintaining intracellular redox balance^18, 19^. Hence, the TrxR has emerged as potential target for anticancer drug design. Selenocystine (SeC) a natural available Se-containing amino acid has been demonstrated effective in inhibiting several cancer cells growth by induction of cell cycle arrest or/and apoptosis through triggering ROS-mediated oxidative damage in our previous studies^5, 11–15^. For instance, SeC can inhibit A549 human lung adenocarcinoma cells growth through inhibition of TrxR activity and TrxR expression *in vitro* and *in vivo* ^20^. However, the antiproliferative activity of SeC against human glioma cells remains elusive, and whether TrxR inhibition was involved in SeC-induced apoptosis in human glioma was not investigated. Hence, in the present study, we evaluated the ability of SeC to inhibit human glioma cells growth *in vitro* and *in vivo*. Mechanistic investigation revealed that SeC effectively suppressed the cells growth and induced apoptosis in human glioma cells by triggering ROS-mediated DNA damage and dysfunction of MAPKs and PI3K/AKT pathways through TrxR1-targeted inhibition *in vitro* and *in vivo*. Our findings validated the strategy to use SeC as a TrxR inhibitor could be a highly efficient way to hunt human glioma.

## Results

### SeC inhibits human glioma cells growth by mitochondria-mediated apoptosis

**Figure 1:**
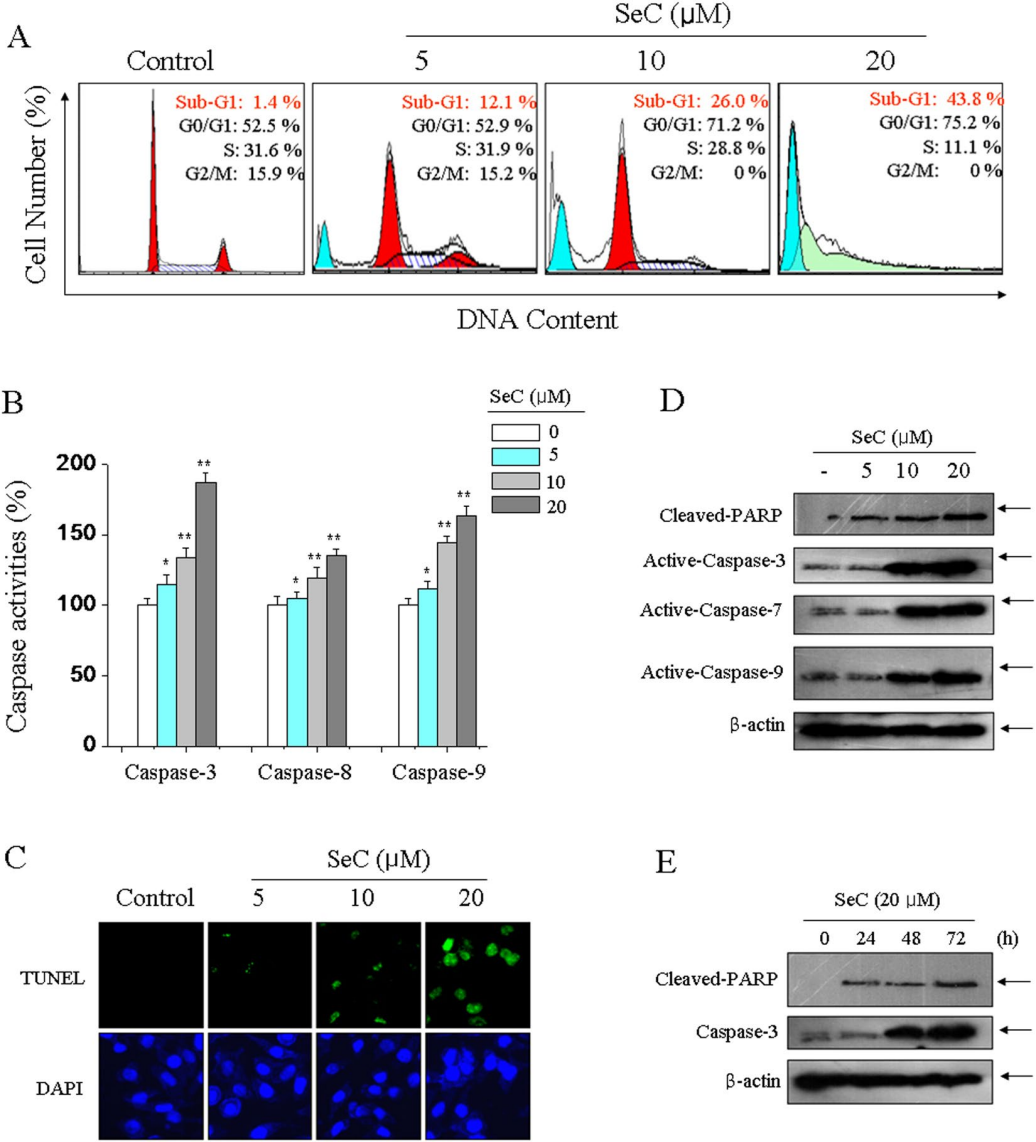
SeC induces apoptosis in human glioma cells. (**A**) Cell apoptosis and cell cycle distribution. U251 cells exposed to SeC were assayed by flow cytometric analysis for cell apoptosis and cell cycle distribution. The hypodiploid DNA content (Sub-G1 peak) were considered as the apoptotic cell death. (**B**) Activation of caspases. U251 cells exposed to SeC were collected and total protein was extracted and incubated with specific caspase substrates for examination of caspase activity as described in method section. (**C**) DNA fragmentation. U251 cells exposed to SeC was imaged by TUNEL-DAPI staining. Dose- (**D**) and time-dependent (**E**) effects of SeC on caspases activation and PARP expression. The expression of caspases and PARP was detected by western blotting methods. All data and images are showed with three independent experiments. Bars with “*” or “**” indicate the statistically different at the *P* < 0.05 and *P* < 0.01 level, respectively.

**Figure 2:**
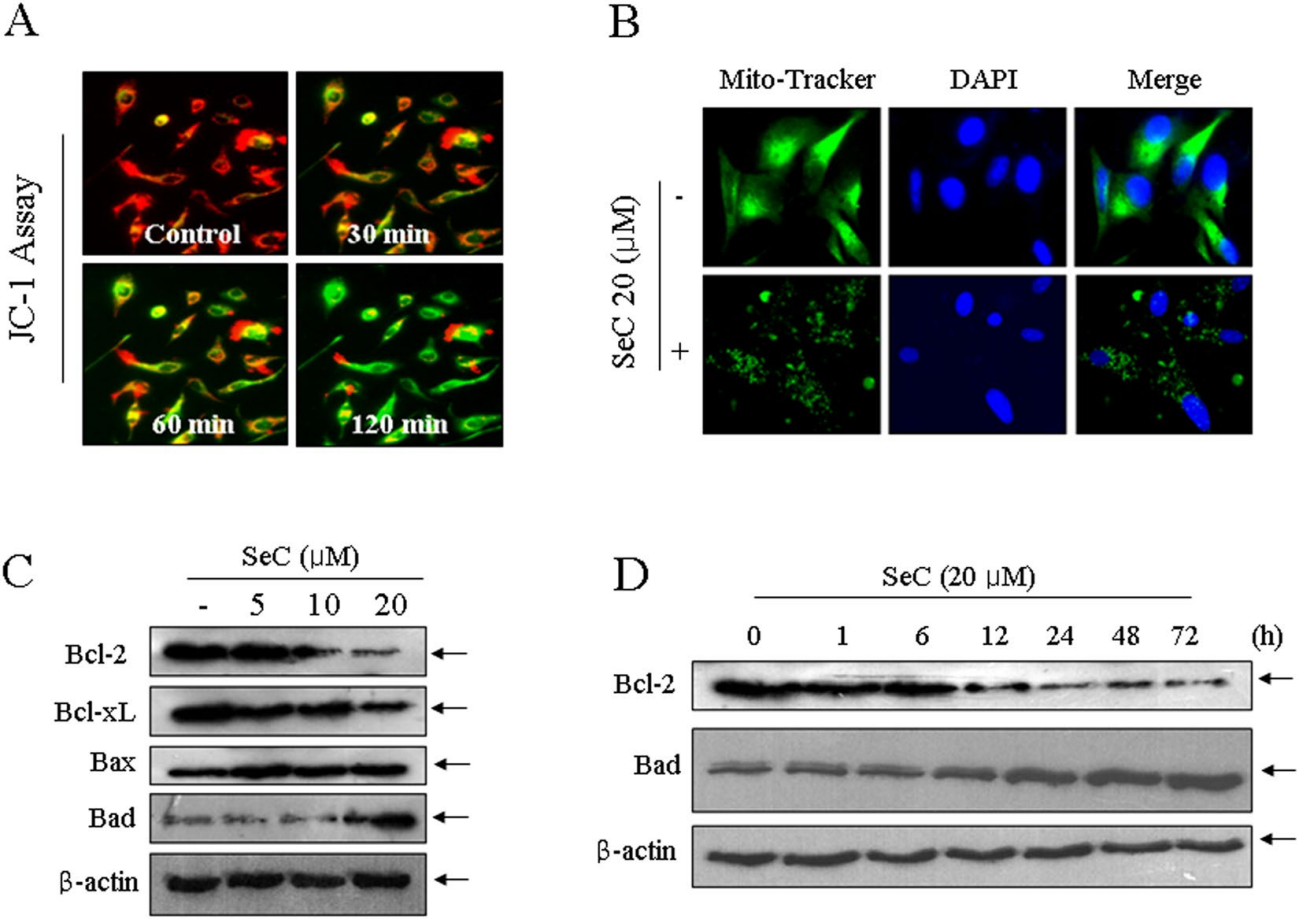
SeC causes the mitochondrial dysfunction via affecting the Bcl-2 family. SeC caused the loss of ∆ψ_m_ (**A**) and mitochondrial fragmentation (**B**). The ∆ψ_m_ and the mitochondrial morphology in living cells were vividly detected by JC-1 and Mitro-tracker probes, respectively. Dose- (**C**) and time-dependent (**D**) effects of SeC on Bcl-2 family expression. The protein expression was examined by western blotting methods. All images here represented three independent experiments with similar results.

Firstly, MTT assay was employed to screen the cytotoxicity of SeC towards human glioma cells. The results showed that SeC treatment significantly inhibited the cell viability of U251 and U87 cells with a dose- and time-dependent manner (Fig. S1). Moreover, SeC treatment showed less cytotoxicity (as measured with MTT assay) towards HUVEC human umbilical vein endothelial cells and BV-2 mouse microglia cells (Fig. S2), indicating the great selectivity of SeC between the normal cells and cancer cells. To explore the cell death mechanism induced by SeC, DNA-flow cytometric analysis and caspase-3 activity were firstly conducted to detect the cell apoptosis. The results showed that SeC treatment both caused significant apoptosis in U251 cells (Fig. 1A) and U87 cells (Fig. S3). For instance, U251 cells exposed to SeC for 72 h showed dose-dependent apoptosis as reflected by the increase of Sub-G1 peak (Fig. 1A). Activation of caspase-3 in SeC-treated U251 cells (Fig. 1B) and U87 cells (Fig. S1C) further confirmed SeC-induced apoptosis in human glioma cells. Moreover, SeC-induced DNA fragmentation was also detected by TUNEL assay, which further vividly validated the apoptotic properities in U251 cells (Fig. 2C). These results above suggest that SeC inhibits human glioma cells growth *in vitro* through induction of apoptosis.

In view of the more cytotoxicity, caspase-3 activation and cell apoptosis in SeC-treated U251 cells, hence, U251 cells was selected for further mechanism evaluation. Primarily, the caspases requirement was examined in U251 cells. As shown in Fig. 1B, SeC treatment dose-dependent activated the caspase-8 and caspase-9, revealing the activation of both mitochondrial- and death receptor-mediated apoptotic pathways. Caspase-9, as the main trigger of mitochondrial-mediated apoptosis, showed more activation than that of caspase-8, indicating the domination of mitochondria-mediated apoptosis. Herein, we interpreted mitochondrial apoptotic markers by western blotting method. As shown in Fig. 1C/D, SeC time- and dose-dependently triggered the activation of caspase-3, caspase-7 and caspase-9. The activation of caspase-7/9 subsequently caused PARP cleavage, and eventually initiated mitochondrial-mediated apoptosis. Taken together, these results clearly indicated that SeC induced mitochondria-mediated apoptotic pathway in U251 cells.

### SeC causes mitochondrial dysfunction by regulating Bcl-2 family expression

Based on the significance of mitochondria in regulating mitochondrial-mediated apoptotic pathway, the mitochondrial membrane potential (Δ*ψm*) and the mitochondrial morphology were both examined. As expected, the loss of Δ*ψm* as an early apoptotic event was obviously observed as early as in 2 h by JC-1 probe, as depicted by the fluorescence shift from red to green in SeC-treated U251 cells (Fig. 2A). Moreover, SeC treatment also caused mitochondrial fragmentation. As shown in Fig. 2B, health U251 cells showed filamentous mitochondrial network with extensively interconnection throughout the cytoplasm. SeC treatment dramatically caused the mitochondrial fragmentation from protonema to punctiform. These findings clearly suggested that SeC caused mitochondrial dysfunction in U251 cells.

Bcl-2 family, including the pro-apoptotic and pro-survival members, has been identified as essential factors in regulating the mitochondrial permeability^21, 22^. Therefore, it is of great significance to detect whether the imbalance of Bcl-2 family was involved in SeC-induced mitochondrial dysfunction. As shown in Fig. 2C, SeC treatment dose-dependently suppressed the Bcl-2 and Bcl-XL expression, but increased the expression of Bax and Bad. The time-course showed that SeC caused continuous down-regulation of Bcl-2 and up-regulation of Bad at the point of 12 h. These results above suggested that SeC induced mitochondria-mediated apoptosis by triggering mitochondrial dysfunction through affecting Bcl-2 family balance.

### SeC triggers ROS-mediated DNA damage

**Figure 3:**
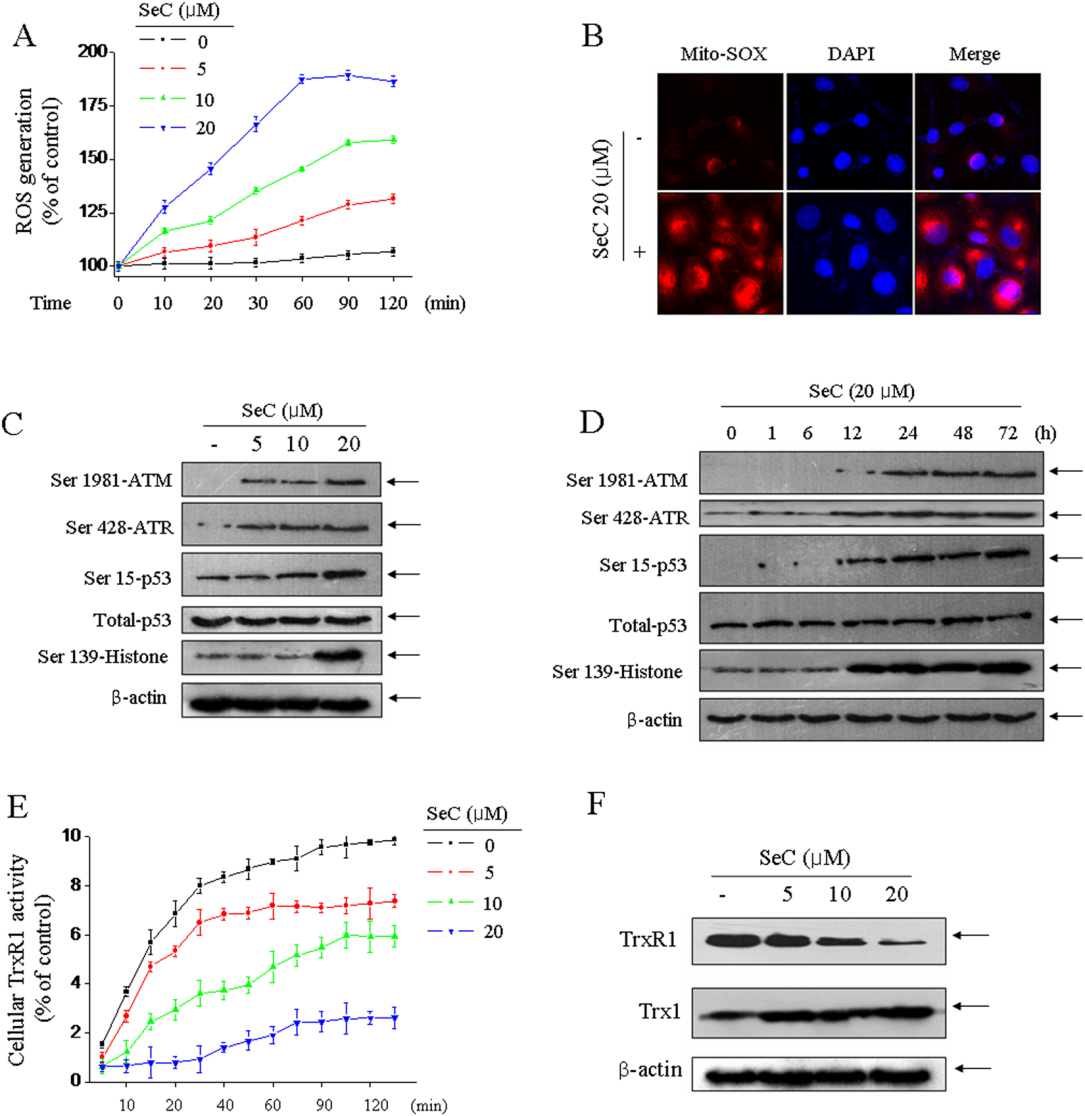
SeC triggers ROS-mediated DNA damage through TrxR1-targeted inhibition. (**A**) Kinetics effect of SeC on ROS generation. U251 cells after pre-incubation with DCFH-DA probe for 30 min were washed and suspended into 10^7^ cells/ml, and incubated with various concentration of SeC. The intracellular ROS generation in living cells was real-timely measured by fluorescence microplate reader. (**B**) Mitochondrial superoxide anion. Cells after treatment with SeC for 2 h were pre-incubated with Mito-SOX for detection of superoxide anion. Dose- (**C**) and time-dependent (**D**) effects of SeC on DNA damage signal axis. The protein expression was examined by western blotting methods. (**E**) Kinetics effect of SeC on TrxR1 activity. The intracellular total protein extracted from normal U251 cells was incubated with or without SeC *in vitro*. Then, the reduction reaction was started after supplement of NADPH and DNTB. The TrxR1 activity was real-time examined by measuring the TNB^2−^ content at 412 nm. (**F**) SeC inhibited TrxR1 expression. The TrxR1 expression was investigated by western blotting. All data images are showed with three independent experiments.

Previous studies have found that SeC inhibited human glioma cells growth in 48 h mainly by induction of S-phase arrest through triggering ROS-mediated DNA damage^5^. To explore the oxidative status in SeC-induced apoptosis, we subsequently investigated the ROS generation and several oxidative damage markers. As show in Fig. 3A, SeC treatment resulted in time- and dose-dependent increase of ROS accumulation as early as in 10 min. Meanwhile, SeC treatment also caused the superoxide overproduction detected by MitoSOX, a specific mitochondria-targeted probe (Fig. 3B). ROS can cause DNA damage, which is accepted as one of the most effective ways in hunting human cancers. Hence, the DNA damage signal axis was examined. The results showed that SeC treatment triggered significant activation of DNA damage signal axis with a dose- and time-dependent manner, as revealed by the continuous phosphorylation of histone (Ser139), p53 (Ser15), total-p53, ATR (Ser428) and ATM (Ser1981) (Fig. C/D). Taken together, these results suggested that ROS-mediated DNA damage contributed to SeC-induced apoptosis in U251 cells.

### TrxR1-targeted inhibition by SeC

Previous studies have revealed that SeC induced oxidative damage-mediated apoptosis in A549 human lung cancer cells by TrxR1-targeted inhibition^20^. To clarify the possibility, TrxR1-targeted inhibition was also detected in U251 cells by a TrxR1 activity detecting kit. As shown in Fig. 3E, after incubation of total protein with SeC, the TrxR1 activity showed apparent inhibition in time- and dose-dependent manner. This inhibitory effect of SeC on TrxR1 activity was measured as early as in 10 min. Furthermore, SeC treatment also decreased TrxR1 expression, followed by an increase of Trx1 expression (Fig. 3F). Based on these results above, we speculated the possibility that SeC treatment disturbed the redox homeostasis through TrxR1-targeted inhibition, caused ROS accumulation and oxidative damage, and ultimately induced human glioma cells apoptosis.

### SeC disturbs the MAPKs and AKT pathways

**Figure 4:**
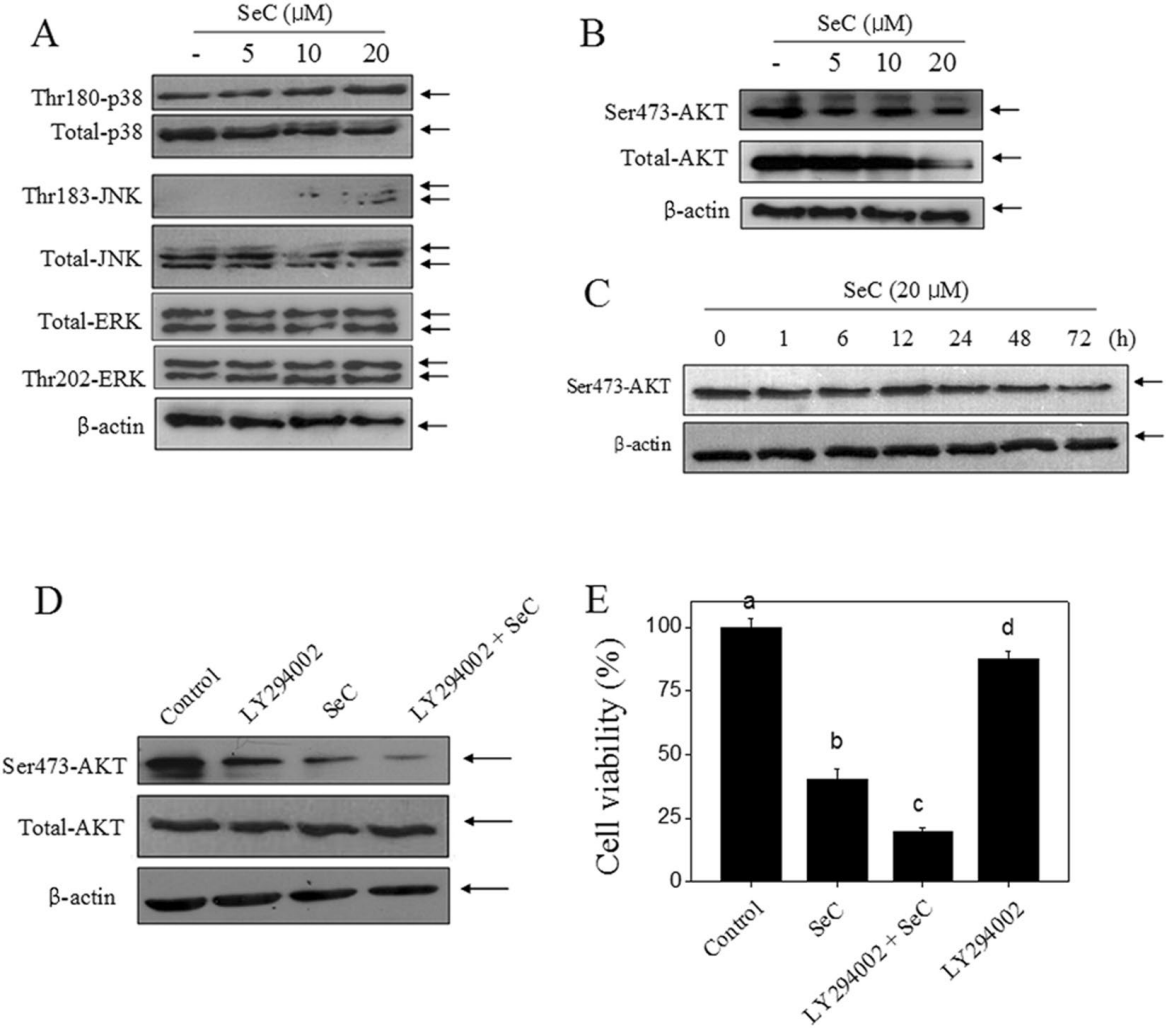
SeC disturbs the MAPKs and AKT pathways. (**A**) Effect of SeC on MAPKs pathway. Dose- (**B**) and time-dependent (**C**) effects of SeC on Ser473-AKT expression. Cells were treated with SeC for 72 h, and the protein expression was analyzed by western blotting methods. Effects of LY294002 (AKT upstream inhibitor) on AKT expression (**D**) and cell viability (**E**) in U251 cells. Cells were pre-treated with LY294002 (AKT upstream inhibitor) for 2 h and co-treated with SeC for 72 h. The protein expression and cell viability were detected by western blotting method, respectively. All images here represented three independent experiments with similar results.

MAPKs and PI3/AKT pathways both play key role in cancer cells survival and tumor resistance in response to chemotherapeutic drugs^23–26^. Increasing evidences have proved that p53 signal and Bcl-2 family both correlated MAPKs and PI3/AKT pathways in drugs-induced cancer cells apoptosis^14, 15, 20^. Herein, we evaluated the status of MAPKs and PI3/AKT pathways in SeC-treated U251 cells. As shown in Fig. 4A, SeC treatment dose-dependently increased the phosphorylated level of JNK (Thr183) and p38 (Thr180), no significant change of p-ERK (Thr202) was detected. However, SeC treatment caused continuous inactivation of p-AKT (Ser473) in a dose- and time-dependent manner (Fig. 4B/C). Additionally, three protein kinase inhibitors, SB202190 (p38 inhibitor), SP600125 (JNK inhibitor) and LY294002 (AKT upstream inhibitor) were employed to further character the role of MAPKs and PI3/AKT pathways in SeC-induced apoptosis and cell growth inhibition. As shown in Fig. 4D/E, pre-treatment with LY294002 significantly enhanced SeC-induced AKT inactivation and cytotoxicity in U251 cells. However, pre-treatment of cells with SB202190 or SP600125 significantly inhibited SeC-induced cytotoxicity against U251 cells (Fig. S4). These findings validated that MAPKs and PI3/AKT pathways both contributed to SeC-induced cell growth inhibition and apoptosis in U251 cells.

### SeC causes ROS-dependent DNA damage and apoptosis

**Figure 5:**
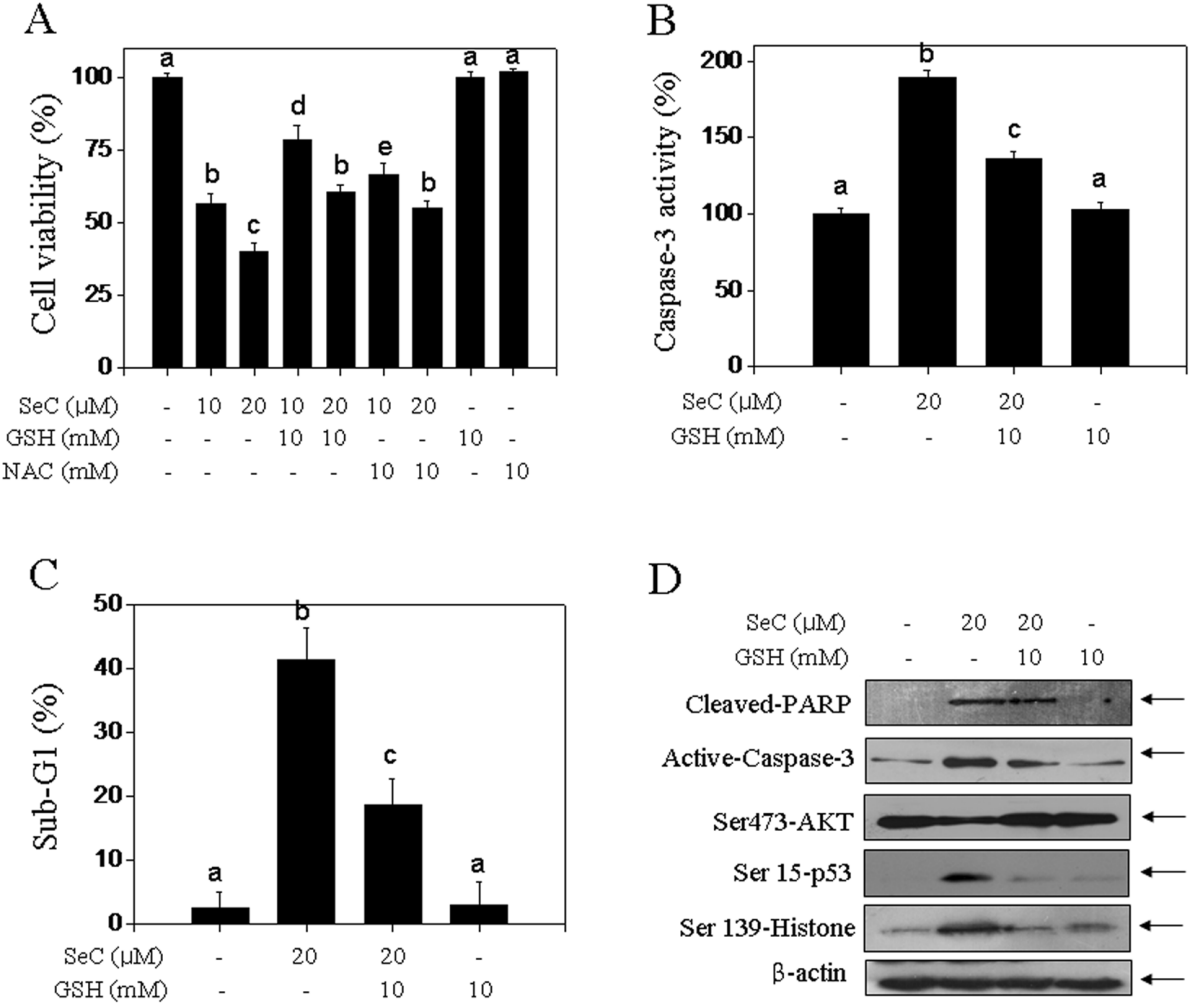
Role of ROS in SeC-induced DNA damage and apoptosis. Supplement of antioxidants blocked U251 cells from SeC-induced cell killing (**A**), caspase-3 activation (**B**) and apoptosis (**C**). Cells were pretreated with 2 mM GSH or NAC for 2 h before SeC co-treatment. The cell viability, caspase-3 activity and apoptosis were detected by MTT assay, fluorometric analysis and flow cytometry, respectively. (**D**) GSH supplement inhibited SeC-induced apoptosis, DNA damage and AKT inactivation. The protein expression was measured by western blotting. All data was expressed as mean ± SD from three independent experiments. Bars with different characters indicates the statistical different at *P* < 0.05 level.

Based on the importance of ROS in SeC-induced DNA damage and apoptosis, we employed the ROS inhibitors, glutathione (GSH) and N-acetylcysteine (NAC), two thiol-reducing antioxidants. Firstly, pre-treatment with GSH or NAC both significantly attenuated SeC-induced cell growth inhibition against U251 cells (Fig. 5A). Secondly, SeC-induced apoptosis was also effectively inhibited after GSH pre-treatment, as proved by the suppression of caspase-3 activity (Fig. 5B) and Sub-G1 peak (Fig. 5C). Moreover, several molecular markers after ROS inhibition in SeC-treated U251 cells were examined. As shown in Fig. 5D, ROS inhibition by GSH distinctly attenuated PARP cleavage, caspase-3 activation, recovered AKT phosphorylation (Ser473) and inhibited the phosphorylation of p53 and histone, indicating that ROS inhibition effectively inhibited SeC-induced DNA damage and apoptosis. These results also verified the possibility that ROS as upstream modulator was involved in SeC-induced DNA damage and apoptosis. We speculated that supplement of thiol-reducing antioxidants, GSH and NAC, not only eliminated the intracellular free radical, but also replenished the intracellular endogenous antioxidants, and eventually reversed SeC-induced DNA damage and apoptosis. Taken together, these results suggested that SeC induced ROS-mediated DNA damage and apoptosis with a ROS dependent manner.

### SeC inhibits U251 tumor xenografts growth ***in vivo***

**Figure 6:**
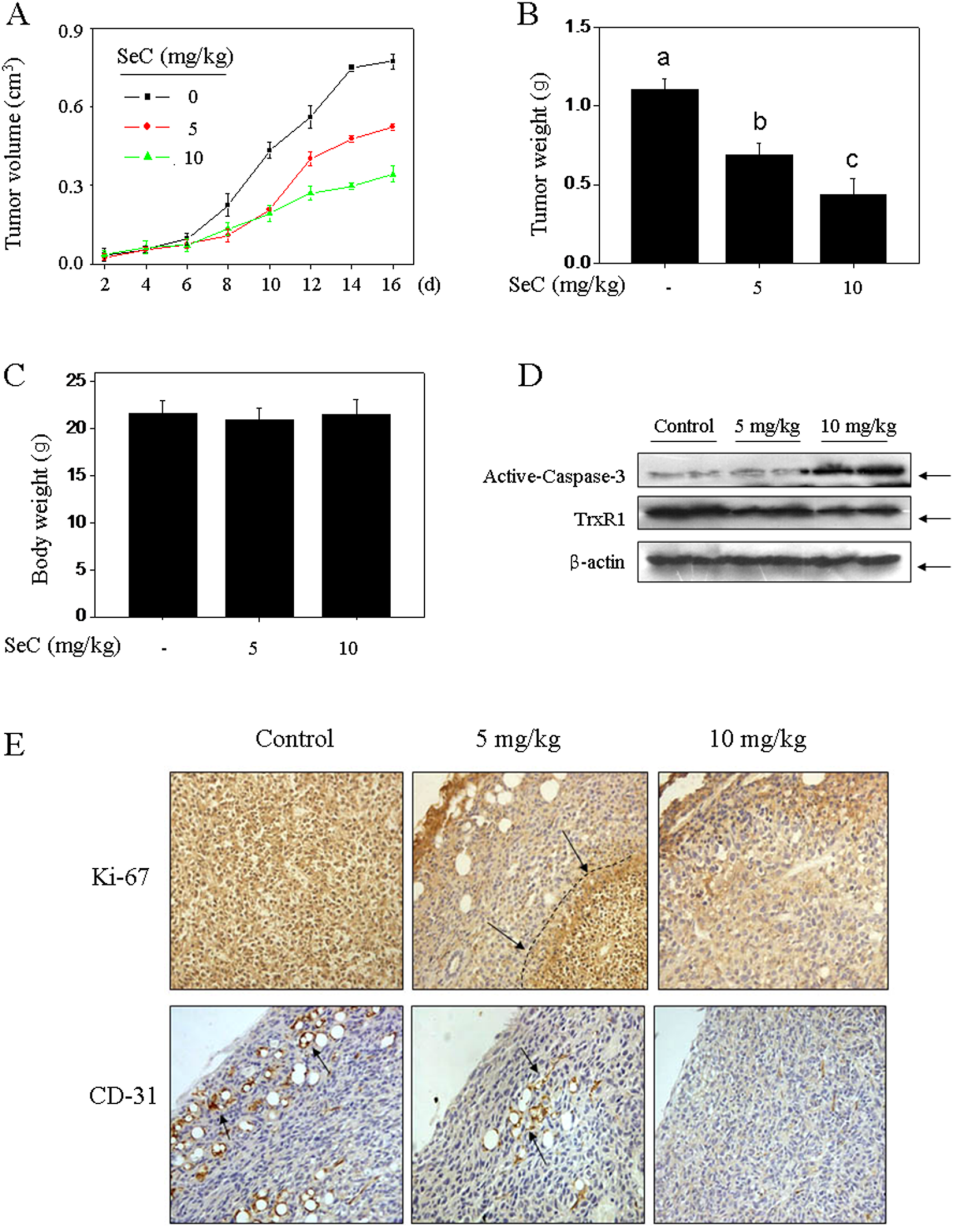
SeC inhibits U251 tumor xenografts growth *in vivo*. SeC blocked the tumor volum (**A**), tumor weight (**B**), but not affected the mice body weight (**C**). SeC (5 and 10 mg/kg) was administrated subcutaneously every other day for 16 days (8 times). (**D**) SeC activated caspase-3 and inhibited TrxR1 expression *in vivo*. (**E**) SeC abrogated the cell proliferation and angiogenesis *in vivo*. The cell proliferation and angiogenesis were observed by Ki-67 and CD-31 staining, respectively. The arrows in anti-Ki-67 image indicate the boundary between Ki-67 high expression and Ki-67 low expression. The arrows in anti-CD-31 image indicate the vascular endothelial cells. All data was expressed as mean ± SD from three independent experiments. Bars with different characters indicates the statistical different at *P* < 0.05 level.

To confirm the *in vivo* inhibitory effect, the immuno-deficient nude mice were employed. After 16-day’s treatment (8 times) with SeC (5 and 10 mg/kg), the tumor weight and tumor volume were both inhibited (Fig. 6A/B). No significant change in the body weight was detected among the groups (Fig. 6C). Additionally, the *in vivo* anticancer mechanism was also investigated. As show in Fig. 6D/E, SeC administration *in vivo* significantly inhibited the cell proliferation (Ki-67 staining), suppressed the tumor angiogenesis (CD-31 staining) and induced apoptosis (caspase-3 activation). For instance, the arrows in anti-Ki-67 image indicate the boundary that the area of Ki-67 high expression and area of ki-67 low expression. The arrows in anti-CD-31 image indicate the vascular endothelial cells. That is, after treatment with SeC, the tumor area became normalization, especially the circum of the tumor. Importantly, the TrxR1 expression *in vivo* was also inhibited by SeC treatment which consists with the results *in vitro*. These results revealed that SeC inhibited human glioma cell growth by induction of apoptosis with involvement of TrxR1 inhibition *in vivo*.

## Discussion

Resistance of human malignant glioma to conventional chemotherapy remains one of the biggest challenges for therapy failure in clinic. Hence, searching agents with high efficiency and low side effects has always attracted much attention of scientists in the medical and pharmaceutic fields. In the present study, we demonstrated the ability of SeC to inhibit human glioma cell growth *in vitro and in vivo* by oxidative damage-mediated apoptosis through TrxR1-targeted inhibition. Our findings validated the potential of Se-containing compounds as tumor chemotherapeutic agent in combating human malignant glioma.

Induction of cancer cell apoptosis was the major action mechanism by Se-containing compounds^7^. Extrinsic (death receptor-mediated) and intrinsic (mitochondria-mediated) apoptotic pathways both contributed to Se-containing compounds-induced cancer cell apoptosis^27^. Previous studies have illuminated the anticancer mechanism of Se-containing compounds, including PARP cleavage, caspase activation, mitochondrial dysfunction, imbalance of Bcl-2 family expression, ROS generation, oxidative damage and dys-regulation of MAPKs and PI3K/AKT pathways^12–15, 20, 27^. In the present study, PARP cleavage, activation of caspase-3, -7, -8, and -9, mitochondrial morphological changes and loss of mitochondrial membrane potential, imbalance of Bcl-2 family expression, overproduction of ROS and superoxide anion, DNA damage and p53 phosphorylation (Ser15), dys-regulation of MAPKs and PI3K/AKT pathways were all detected after SeC treatment in human glioma cells, which suggested the consistency of Se-containing compounds-induced apoptosis. Mitochondria as the main organelle in regulating cell apoptosis process plays key role in Se-containing compounds-induced cancer cells apoptosis. Regulation of mitochondrial membrane potential by Bcl-2 family was considered as potential factors in regulating Se-containing compounds-induced cell apoptotic death^21, 22, 28–31^. In the present study, SeC treatment reduced Bcl-2 and Bcl-XL expression, but significantly increased Bax and Bad expression. The imbalance of Bcl-2 family by SeC certainly lead to the dysfunction of mitochondria, such as the loss of mitochondrial membrane potential and changes of mitochondrial morphology. These results indicated that SeC triggered mitochondria-mediated apoptosis by regulating Bcl-2 family in human gloma cells.

Induction of ROS-mediated oxidative damage to inhibit human cancer cells growth represents one of the important strategies in clinic. Previous studies also supported that ROS-mediated oxidative damage was involved in Se-containing compounds-induced tumor cells apoptosis^12–15^. Se-containing compounds as double-edged agents usually display pro-oxidant property against human cancer cells. It is reported that SeC showed novel anticancer activity against several human tumor cells by induction of ROS-mediated oxidative damage and apoptosis^12–15^. Meanwhile, inhibition of ROS effectively blocked Se-containing compounds-induced oxidative damage and cancer cell apoptosis, indicating the important role of ROS in Se-containing compounds-mediated anticancer mechanism in human tumors^12–15^.

TrxR as a key regulator always maintains the intracellular redox balance. TrxR-based anti-oxidative system was overexpressed in many human cancer cells, which constitutes the main defensive system against exogenous stimuli^18, 19^. Hence, dysfunction of TrxR function or/and inhibition of TrxR activity both represent novel strategies for therapy of human cancers, and TrxR is emerging as potential target for anticancer drugs design^32–40^. ROS-mediated oxidative damage is considered as the main apoptotic events by Se-containing compounds^10, 12, 13, 15^. However, the specific producing mechanism of free radical and the disorder of intracellular antioxidant system by Se-containing compounds have not been well explored yet. TrxR contains a selenocysteine residue and selenium supplement can affect the TrxR-mediated redox regulation. We have already proved that SeC could affect TrxR activity and expression, and cause oxidative damage-mediated apoptosis in A549 human lung adenocarcinoma cells^20^. Whether SeC can affect the TrxR activity or/and expression in human glioma cells has not been well explored yet. In the present study, SeC significantly induced intracellular ROS overproduction by inhibiting TrxR1 activity and expression, and subsequently triggered oxidative damage and apoptosis in human glioma cells. Addition of antioxidants (such as NAC and GSH) reversed SeC-induced oxidative damage and apoptosis in human glioma cells. These results revealed that SeC can act as a TrxR1-targeted inhibitor to hunt human glioma growth.

In summary, we demonstrated the potential of SeC to inhibit human glioma cells growth *in vitro* and *in vivo* by oxidative damage-mediated apoptosis through TrxR1-targeted inhibition. Our findings validated the strategy to use TrxR1-targeted inhibition by SeC could be a highly efficient way to combat human glioma growth.

## Methods

### Cell culture and cell viability assay

U251 and U87 human glioma cells were purchased from ATCC company (USA). Cells were cultured with DMEM medium added with 10% FBS and maintained in 5% CO_2_ at 37 °C. Cell viability was detected by MTT assay. Briefly, U251 or U87 cells (6 × 10^3^ cells/well) seeded in 96-well plate were treated with 0–40 μM SeC for 24, 48 and 72 h. After treatment, 20 μl of MTT solution was added and incubated at 37 °C for 5 h. After incubation, the supernatants were replaced with 150 μl DMSO. Then the absorbance measured at 570 nm was employed to reflect the cell growth condition. The cell viability was presented as % of control. All experiments were conducted in accordance with the relevant guidelines and regulations of Taishan Medical University.

### Cell apoptosis

Cell apoptosis and cell cycle distribution were analyzed by flow cytometric (FCM) analysis. Briefly, U251 cells after treatment with SeC for 72 h were collected, fixed with 70% ethanol and stained by PI solution. After washing with PBS, cell apoptosis was detected by FCM analysis by quantifying the Sub-G1 peak, and the cell cycle distribution was analyzed by the ModFit Software. Per sample about 10^4^ cells were recorded. All experiments were conducted in accordance with the relevant guidelines and regulations of Taishan Medical University.

### Caspases activity, ROS and superoxide anion

Caspases activity in U251 cells was examined by three specific substrates, including Ac-DEVD-AMC (caspase-3), AC-LEHD-AMC (caspase-9) and Ac-IETD-AMC (caspase-8). The kinetics course of ROS generation in U251 cells was monitored using a DCFH-DA probe in 2 h. The mitochondrial superoxide anion in living U251 cells was vividly imaged by Mito-SOX, a mitochondria-targeted red probe. The experimental details of the three detective methods were carried out according to our previous report^5^. All experiments were conducted in accordance with the relevant guidelines and regulations of Taishan Medical University.

### TUNEL-DAPI staining

The DNA fragmentation and chromatin condensation were detected by TUNEL-DAPI kit. Briefly, U251 cells after treatment were fixed with paraformaldehyde and permeabilized with Triton X-100. Then cells were incubated TUNEL and DAPI solution, respectively. Cells were imaged by fluorescence microscope and the TUNEL-positive cells (green) indicated the apoptotic cells. The details of the experiment were administrated as previously reported^27^. All experiments were conducted in accordance with the relevant guidelines and regulations of Taishan Medical University.

### Mitochondrial membrane potential (Δψm) and mitochondrial morphology

The intracellular Δψm in living U251 cells was examined by JC-1 probe. Briefly, cells seeded in 2-cm culture plate were incubated with 10 μM JC-1 for 30 min. After incubation, cells were cleaned and photographed at 30, 60 and 120 min for the Δψm after supplement with SeC. The mitochondrial morphology was observed by Mito-Tracker and DAPI co-staining. All experiments were conducted in accordance with the relevant guidelines and regulations of Taishan Medical University.

### Western blotting

RIPA lysis buffer was used to separate the intracellular total protein. The concentration of total protein was quantified by BCA quantitation kit. Total protein (40 μg/lane) after denaturation was separated by SDS-PAGE (10%) and transferred onto PVDF membranes. The membrane after blocking with 5% non-fat milk was subsequently incubated with the primary and second antibodies, respectively. After washing with TBST, the protein was imaged by an enhanced ECL system. The experimental details of the detective methods were carried out according to our previous report. All experiments were conducted in accordance with the relevant guidelines and regulations of Taishan Medical University.

### TrxR1 activity

Firstly, the cytosolic protein was prepared and quantified by BCA kit. Then the TrxR1 activity was examined with the TrxR kit. The fresh protein (100 μg/well) diluted with buffer was added into the 96-well plate and incubated with or without SeC for 10 min at 37 °C. After incubation, the reaction was started after supplement of NADPH and DNTB. Immediately, the kinetics effect of SeC on TrxR1 activity in 2 h was real-time monitored at 412 nm. The data was expressed as the percentage of control. All experiments were conducted in accordance with the relevant guidelines and regulations of Taishan Medical University.

### *In vivo* study

The *in vivo* anticancer effect of SeC on human glioma was evaluated in BALB/cA nude mice. Briefly, 5 × 10^6^ U251 cells diluted in 100 μl FBS-free medium were injected into the flank of the mice. After one-week growth, the appropriate mice bearing the tumor volume about 100 mm^3^ were selected for the subsequent experiments. SeC (5 and 10 mg/kg) was injected from the caudal vein every other day (about 8 times). At the termination of the experiments, the tumor volume, tumor weight and the body weight of mice were all recorded. For mechanism investigation, portion of the tumor tissue was employed for immunohistochemistry (IHC) and western blotting assay, respectively. All animal experiments were approved by the Institutional Animal Ethics Committee of Taishan Medical University.

### Statistical analysis

All data was presented as mean ± SD which was obtained from three independent experiments. The statistical analysis was carried out by SPSS013.0 software. The significance between two groups was analyzed by two-tailed Student’s test. The difference among three or more groups was analyzed by multiple comparisons. Bars “*” or “**” represent the *P < *0.05 or *P < *0.01, respectively. Bars with different characters indicates the statistical different at *P* < 0.05 level.

## Electronic supplementary material

Supplementary Information
