# Supplementary material for: Selenocysteine induces apoptosis in human glioma cells: evidence for TrxR1-targeted inhibition and signaling crosstalk
Source: Sci Rep. 2017 Jul 25;7:6465. doi: 10.1038/s41598-017-06979-2 (PMC5526989; doi:10.1038/s41598-017-06979-2)
Supplement: Supplementary file 1 — Supplementary Information [file 41598_2017_6979_MOESM1_ESM.pdf]

Supporting Information

for

Selenocysteine induces apoptosis in human glioma cells: evidence for TrxR1-targeted inhibition and signaling crosstalk

Cun-dong Fan[1], Xiao-yan Fu[1], Zong-yong Zhang[1], Ming-zhi Cao[2], Jing-yi Sun[3], Ming-feng Yang[1], Xiao-ting Fu[1], Shi-jun Zhao[1], Lu-rong Shao[1], Hui-fang Zhang[1], Xiao-yi Yang[1*], Bao-liang Sun[1,4*]

[1] Key Lab of Cerebral Microcirculation in Universities of Shandong, Taishan Medical University, Taian, Shandong, 271000, China

[2] Department of Neurosurgery, Huxi Hospital, Jining Medical University, Shanxian 274300, Shandong, China

[3] Wonju Severance Christian Hospital, Yonsei University Wonju College of Medicine, Wonju, Gangwon 220-701, Korea

[4] Department of Neurology, Affiliated Hospital of Taishan Medical University, Taian 271000, Shandong, China

Cun-dong Fan, Xiao-yan Fu and Zong-yong Zhang contributed equally to this work.

*** Corresponding authors.**

Bao-liang Sun, Yingsheng East Road 2, Taishan Medical University, Taian, Shandong, 271000, China. Email: tblsun66@163.com, Tel: +86-538-6230030, Fax: +86-538-6230030

Xiao-yi Yang, Yingsheng East Road 2, Taishan Medical University, Taian, Shandong, 271000, China. Email: xyyang@tsmc.edu.cn, Tel: +86-538-6230027, Fax: +86-538-6230027

Running title: Selenocysteine induces apoptosis in human glioma cells

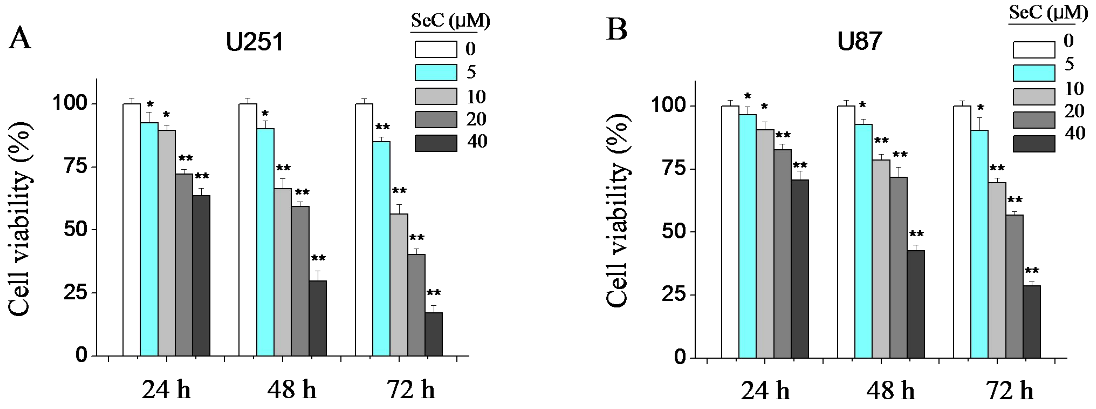

Fig. S1. Cytotoxicity of SeC towards human glioma cells. Growth inhibition of SeC against U251 (A) and U87 cells (B). U251 or U87 cells (6×10^3 cells/well) were seeded in 96-well plate and pre-incubated for 24 h. Then cells were treated with 0-40 μM SeC for 0-72 h. Cell viability was detected by MTT assay and the result was expressed as percentage of control (as 100%). All data were expressed as mean ± S.D. Bars with "*" or "**" indicate the statistically different at the $P<0.05$ and $P<0.01$ level, respectively.

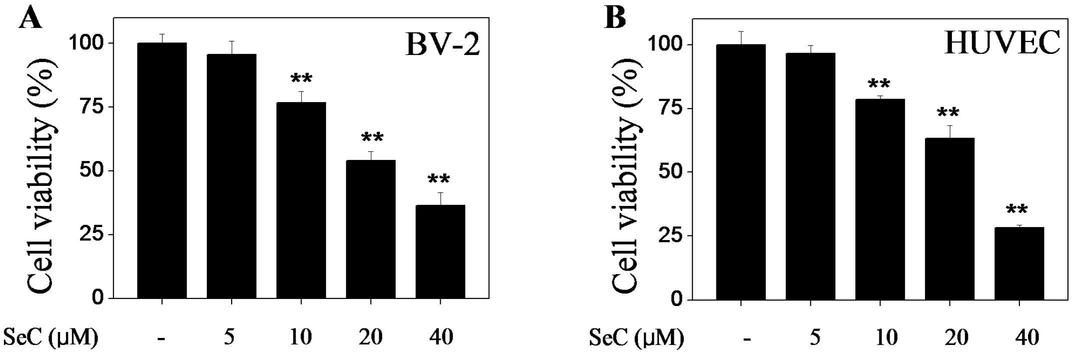

Fig. S2. Cytotoxicity of SeC towards human normal cells. Growth inhibition of SeC against BV-2 mouse microglia cells (A) and HUVECs human umbilical vein endothelial cells (B). BV-2 or HUVECs cells (6×10^3 cells/well) were seeded in 96-well plate and pre-incubated for 24 h. Then cells were treated with 0-40 μM SeC for 72 h. Cell viability was detected by MTT assay and the result was expressed as percentage of control (as 100%). All data were expressed as mean ± S.D. Bars with "**" indicate the statistically different at the $P<0.01$ level.

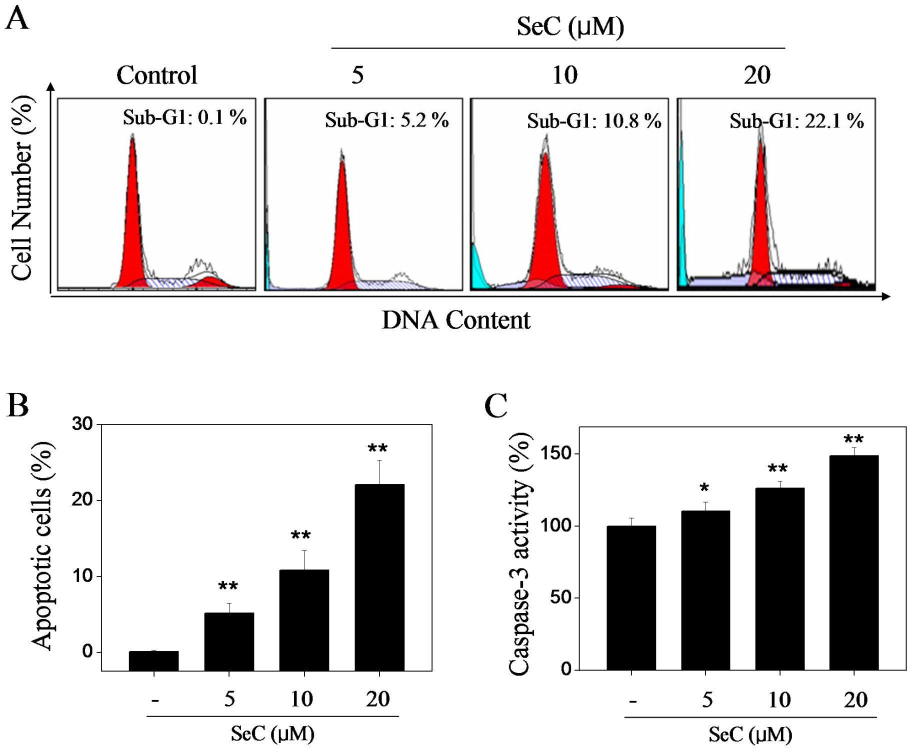

Fig. S3. SeC induces apoptosis in U87 cells. (A) SeC induces apoptosis in U87 cells. (B) Statistic analysis of apoptotic cells. (C) Caspase-3 activity. Cells were treated with 0-20 μM SeC for 72 h. After treatment, cells were collected and detected for apoptosis and caspase-3 activity as described in section of method. All data and images are showed with three independent experiments. All data were expressed as mean ± S.D. Bars with "*" or "**" indicate the statistically different at the P<0.05 and P<0.01 level, respectively.

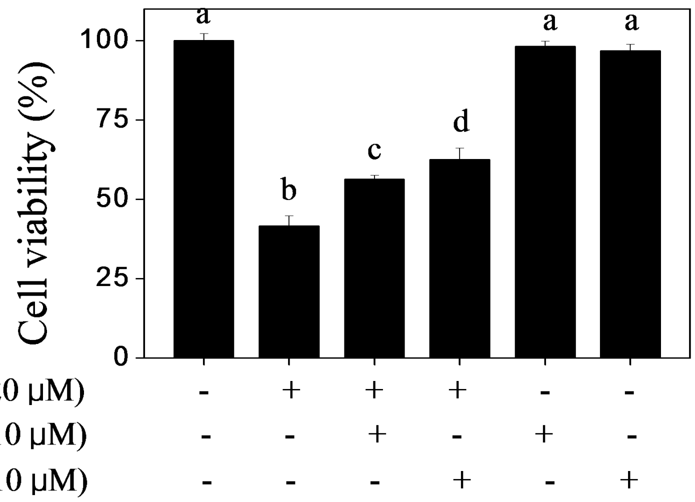

Fig. S4. Effects of kinase inhibitors on cell viability in SeC-treated U251 cells. The cells were pre-treated with 10 μM SB202190 (p38 inhibitor) or SP600125 (JNK inhibitor) for 2 h before SeC treatment. All data here are expressed as means ± SD of triplicates. The image shown here is representative of three independent experiments with similar results. Bars in the figures with different characters are statistically different at the $P<0.05$ level.
